# Microbiological Testing for the Proper Assessment of the Hygiene Status of Beef Carcasses

**DOI:** 10.3390/microorganisms7030086

**Published:** 2019-03-19

**Authors:** Anderson Carlos Camargo, Marcus Vinícius Coutinho Cossi, Wladimir Padilha da Silva, Luciano dos Santos Bersot, Mariza Landgraf, József Baranyi, Bernadette Dora Gombossy de Melo Franco, Nero Luís Augusto

**Affiliations:** 1 Universidade Federal de Viçosa, Departamento de Veterinária, Campus UFV, Viçosa 36570-900, MG, Brazil; anderson.c.camargo@hotmail.com; 2 Universidade Federal de Uberlândia, Faculdade de Medicina Veterinária, Campus Umuarama, Uberlândia 38400-902, MG, Brazil; marcuscossi@yahoo.com.br; 3 Universidade Federal de Pelotas, Departamento de Ciência e Tecnologia Agroindustrial, Campus Capão do Leão, Pelotas 96001-970, RS, Brazil; wladimir.padilha2011@gmail.com; 4 Universidade Federal do Paraná, Departamento de Ciências Veterinárias, Rua Pioneiro, 2153, Palotina 85950-000, PR, Brazil; lucianobersot@gmail.com; 5 Food Research Center, Universidade de São Paulo, Faculdade de Ciências Farmacêuticas, Departamento de Alimentos e Nutrição Experimental, Cidade Universitária, São Paulo 05508-080, SP, Brazil; landgraf@usp.br (M.L.), bfranco@usp.br (B.D.G.d.M.F.); 6 University of Debrecen, Institute of Nutrition, Böszörményi út 138, H-4032 Debrecen, Hungary; jozsef.baranyi@gmail.com

**Keywords:** cattle slaughterhouses, hygiene monitoring, microbiological criteria, quality control program

## Abstract

Microbiological testing is an important quality management tool in the food industry. In this study, the hygiene status of beef carcasses sampled in eight Brazilian slaughterhouses was assessed by enumeration of different hygiene indicator microorganisms, and a model to establish potential associations among these counts was proposed. The carcasses (*n* = 464) were surface sampled at four slaughtering steps (step 1: Hide after bleeding; step 2: Carcass after hide removal; step 3: Carcass after evisceration; step 4: Carcass after end washing) and subjected to a counting of mesophilic aerobes (MA), Enterobacteriaceae (EB), total coliforms (TC), and *Escherichia coli* (EC) using Petrifilm™ plates. Among the sampled beef carcasses (step 4), 32 (6.9%) and 71 (15.3%) presented counts above the microbiological criteria established by (EC) No. 1441/2007 for MA and EB, respectively. Thus, indicating that improvements in slaughter hygiene and a review of process controls are demanded in some of the studied slaughterhouses. The log count differences of EC, TC, and EB from MA were considered as response variables as a function of the slaughtering steps. Differential log counts changed consistently with the steps. The measurements, including the patterns in their inherently random variability, were fairly predictable from steps 1 and 4. The results indicated that differential log counts for TC and EC are not relevant, as their concentrations and random pattern can be inferred from counts of MA and EB. The proposed model can be used as a valuable tool for the design and adoption of feasible quality control programs in beef industries. The adoption of such a tool should have a positive contribution on consumers’ health and enhance product quality.

## 1. Introduction

The Brazilian beef industry is largely export oriented with 9.71 million tons of beef produced and 2.03 million tons destined to be exported in 2017 [1]. Brazil is currently the second largest beef producer worldwide, behind the USA, and the second largest beef exporter, behind India [1,2]. To keep this prominent position in the global market, the Brazilian beef industry and local inspection agencies rely on strict quality control measures and programs in compliance with national and international quality and safety regulations [3,4,5].

Quality management programs aim to prevent contaminations and ensure food safety. Good hygiene practices (GHP) are mainly focused on facilities, equipment, utensils, employee training, cleaning, sanitation, storage, distribution, and pest control [6], while the hazard analysis and critical control points (HACCP) focus on all steps of processing, through monitoring of critical points for contamination and allowing preventive procedures to avoid the hazard [7,8]. Such controls can be guided by microbiological criteria for foods that include safety parameters related to foodborne microbial pathogens and hygiene indicators, such as counts of mesophilic aerobic bacteria, coliforms and Enterobacteriaceae [9,10]. Thus, hygiene indicator microorganisms (HIM) are essential to indicating that GHP and HACCP programs are in place within the industry [6,11,12,13].

Enumeration of HIM provides information regarding general contamination, incipient spoilage, or reduced shelf-life, i.e., utility of the product. The quantitative information provided by these tests can be very useful for trend analysis and verification of process control as microbiological populations at a certain processing step that surpass a reference value or a trend indicate that corrective measures are necessary, which may include improvement of hygiene conditions and a revision of quality management procedures [6,9,14,15].

Self-control programs focus the entire production chain from initial steps to end products [15,16,17]. The microbiological data generated along the production chain are systematically analyzed, generating a valuable database on the prevalent occurrence of different types of indicator microorganisms [6]. Despite the additional cost for companies, generated databases are valuable for trend analyses as they reflect the contamination dynamics in the production chain, enabling enhancements in the control of the microbiological contamination and improving the quality and safety of the end products [6].

Despite having to meet the established criteria for HIM by many importing countries, primarily in the European Union (EU) [18], Brazilian regulation does not establish criteria for mesophilic aerobes and Enterobacteriaceae on beef carcasses for the domestic market so far. Consequently, these groups of microorganisms are hardly monitored in Brazilian beef carcasses since it involves additional costs, such as professional training and laboratorial analysis. The scientific literature on the microbiological status of Brazilian beef carcasses reports data on the prevalence of pathogens such as Shiga toxin producing *Escherichia coli*, *Salmonella* spp., and *Listeria monocytogenes* as well as others [19,20,21,22,23], but little has been reported on HIM. Thus, the objective of this study was to generate data on the hygiene status of beef carcasses sampled in selected Brazilian slaughterhouses inspected by the Brazilian Ministry of Agriculture, and use the microbiological data to propose a mathematical model to indicate potential associations among the populations of HIM. The end goal is to help the meat industry in the design of proper quality control programs.

## 2. Materials and Methods

### 2.1. Selection of the Slaughterhouses

A total of 8 cattle slaughterhouses subjected to the Brazilian inspection service of the Ministry of Agriculture were selected for the study. The slaughterhouses were located in 3 Brazilian states: Minas Gerais (Sl 01, Sl 02, and Sl 03), Paraná (Sl 04, Sl 05, and Sl 06), and Rio Grande do Sul (Sl 07 and Sl 08). The characteristics of the selected slaughterhouses are shown in Table 1.

### 2.2. Sampling

At each visit to the slaughterhouses, 10 carcasses were randomly selected and the same carcass was sampled at 4 steps of the slaughtering process: (1) hide after bleeding, (2) carcass after hide removal, (3) carcass after evisceration, and (4) carcass after end washing. A total of 464 carcasses were sampled: Sl 01: 69, Sl 02: 70, Sl 03: 70, Sl 04: 25, Sl 05: 40, Sl 06: 40, Sl 07: 75, and Sl 08: 75 carcasses.

Surface sampling was done according to the recommendations of the International Organization for Standardization 17604 [24] and EC 1441 [18]. For sampling at steps 1 and 2, four sterile square plastic templates of 100 cm^2^ were placed in the shoulder and chest areas of the animal (two templates each side, total: 400 cm^2^). The templates were placed in adjacent areas, avoiding sampling of the same site. After splitting the carcasses in 2, two templates of 100 cm^2^ were placed in each side (internal and external areas of the thoracic region, steps 3 and 4), completing 400 cm^2^. Each delimited area was swabbed with a sterile Hydrated-Sponge (3M Microbiology, St. Paul, MN, USA), moistened with 10 mL of sterile saline (0.85% *w*/*v*) (Oxoid Ltd., Basingstoke, UK). The four sponges obtained at the same step (1, 2, 3, or 4) were pooled in one sterile plastic bag (Whirl-Pak^®^, Fort Atkinson, WI, USA), and kept at 4 °C until submitted to the microbiological tests (maximum 6 h).

### 2.3. Enumeration of Hygiene Indicator Microorganisms

The bags with the four sponges were stomached with 160 mL of buffered peptone saline (Stomacher 400 Circulator, Seward Ltd, Worthing, UK) for 60 s and the liquid in the bags was subjected to ten-fold dilutions in sterile saline 0.85% (*w*/*v*). The dilutions were plated on Petrifilm™ plates (3M Microbiology): Petrifilm™ AC for mesophilic aerobes (MA), Petrifilm™ EB for Enterobacteriaceae (EB), Petrifilm™ EC for total coliforms (TC), and *E. coli* (EC), and incubated at 35 °C for 24 to 48 h. Plating procedures and counting of colonies were done according to the recommendations of the manufacturer. Results were expressed as log CFU/cm^2^.

### 2.4. Statistical Analysis

The log values of the mean bacterial populations at each sampling step of the 8 slaughterhouses were submitted to regression analysis to check potential trends at the tested steps. The differences (D) between the log counts of each HIM (D_EB_, D_TC_, and T_EC_) and the total aerobic count (MA) for each sampling step were calculated whenever possible, and the resulting values were studied as a function of the location of the slaughterhouse (Brazilian state) and the slaughtering step, using linear regression and ANOVA. The significance level was fixed at *p* < 0.05. Microsoft Excel and its statistical functions were used for these calculations.

## 3. Results and Discussion

During the cattle slaughtering and processing, contamination can occur via slaughter facility and carcasses handling. Based on these key procedures, regulations and guidelines are in place to ensure the quality and safety of the beef distributed to human consumption. In the present study, we accessed the hygiene status in different steps of cattle slaughtering and the quality of beef carcasses (after final washing) in Brazil. The mean populations of MA, EB, TC, and EC in the tested beef carcasses are shown in Table 2. As expected, samples taken at step 1 (hide after bleeding) presented significantly higher populations of the HIM than samples taken during the other three steps (*p* < 0.0001). At step 2 (after hide removal), the populations of all HIM were at least 1.5 log CFU/cm^2^ lower than those observed in the samples taken at step 1. Other studies carried out in Brazilian slaughterhouses reported a similar trend [22,25].

Evisceration played a significant role in carcass recontamination as populations of EB and TC in samples taken at step 3 were higher than in step 2 (*p* < 0.0001, Table 2). The populations at step 4 (after end washing) were significantly lower than at step 1 (*p* < 0.0001). However, populations at step 4 were higher than in step 2, which was similarly observed by Zweifel, et al. [26], suggesting that final washing was not enough to counterpart the contamination that occurred during evisceration.

When geographical locations (Brazilian states) are compared, samples collected in MG presented higher MA populations than samples from the two other states, in all steps (Table 3). The lowest populations of EB (0.8 log CFU/cm^2^) were observed in samples taken in PR at step 2, and the highest (3.6 log CFU/cm^2^) in RS at step 1. Samples from PR presented the lowest populations for EB, TC and EC, regardless the step of sampling. Despite the observed differences on microbial counts, it is important to highlight that all facilities were inspected based on same and identical guidelines determined by the Brazilian inspection service from the Ministry of Agriculture.

Results of ANOVA indicated that the populations of HIM decreased over processing (steps 1 to 4, *p* < 0.005), regardless the geographical location of the slaughterhouse. Comparisons of the populations indicated that when the counts at step 1 were high, the populations remained high in the following steps suggesting that the hygiene status of animal skin prior to slaughtering may plays a role in carcasses contamination. Hide-to-carcasses microbial cross-contamination can occur under commercial conditions [27,28,29,30]. However, [Hauge, et al. [31]] suggested that higher loads of MA and EB found in dirty cattle hides does not impact the hygienic quality of the carcasses, resulting in carcasses with the same quality of those obtained from animals with clean hides. Anyway, it is important to clarify that carcasses contamination may be influenced by other factors, such as design of each facility and training of employees.

Enumeration of HIM on beef carcass has relevance because results correlate with the hygienic conditions during slaughtering [8,32,33]. Usually, microbiological limits are applied for meat, but some countries also stablish criteria for beef carcasses. In Brazil and in the USA, beef carcass safety is assessed based on detection of foodborne pathogens (such as *Salmonella*, *Listeria monocytogenes*, and pathogenic *Escherichia coli*), while counting of HIM is not mandatory [34,35]. However, in Europe, the (EC) No. 1441/2007 sets limits of MA and EB for cattle carcass after dressing (step 4 of the present study): m = 3.5 log CFU/cm^2^ and M = 5.0 log CFU/cm^2^ for MA, and m = 1.5 log CFU/cm^2^ and M = 2.5 log CFU/cm^2^ for EB [5,18]. In the present study, not all the evaluated beef carcasses in step 4 were within the microbial criteria established by (EC) No. 1441/2007 for MA and EB. As expected, most of the samples with counts above the European criteria were obtained from MG state (Table 4).

The hide, hair, hooves, and gastrointestinal tract are the main sources of microorganism in live animals [10,32,36,37]. Carcasses that are not subjected to contact with the hide during skinning or with the fecal content during evisceration typically present low microbial contamination [27]. Bacterial loads on carcasses can be reduced by spraying with organic acids or other approved antimicrobial compounds, but these compounds, routinely used in certain countries, are not allowed in Brazil [37,38,39].

**Figure 1:**
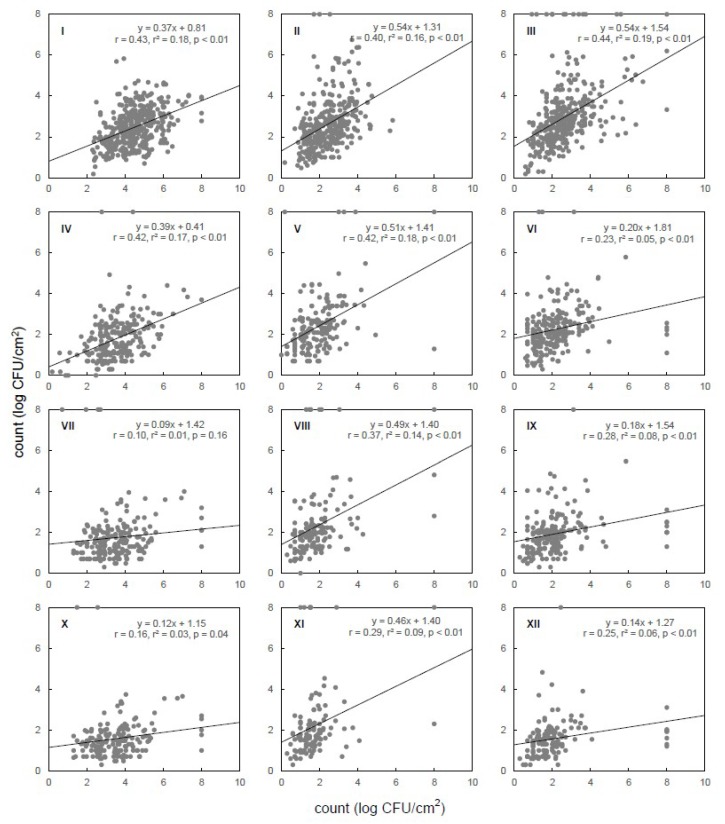
Dispersion of microbial counts (log CFU/cm^2^) of mesophilic aerobes (I, II, III), Enterobacteriaceae (IV, V, VI), total coliforms (VII, VIII, IX), and *Escherichia coli* (X, XI, XII) from bovine carcasses obtained in different steps of slaughtering (‘y’ vs. ‘x’): ‘after hide removal (step 1)’ vs. ‘hide after bleeding (step 2)’ (I, IV, VII, X), ‘after evisceration (step 3)’ vs. ‘after hide removal (step 1)’ (II, V, VIII, XI), and ‘after washing (step 4)’ vs. ‘after evisceration (step 3)’ (III, VI, IX, XII). In each graph, r is the correlation coefficient, r^2^ is the coefficient of determination, and p is the level of significance.

Figure 1 shows the correlation indexes calculated for the microbial counts (log CFU/cm^2^) of MA, EB, TC, and EC in the carcasses obtained at different steps of slaughtering. The correlation indexes ranged from 0.10 for TC to 0.43 for MA when steps 1 and 2 are compared, and from 0.29 for EC to 0.42 for EB when steps 2 and 3 are compared. The indexes between the populations in steps 3 and 4 ranged from 0.23 for EB to 0.44 for MA. These indexes were all significant (*p* < 0.05). Similar trends were observed for samples collected in the three states, though with varying confidence measures (Supplementary Figure S1–3). Such correlation indexes can be useful to find patterns in the contamination levels at different steps of the food processing chain, helping to select the key steps that require extra attention and rigorous control. Although the evaluation of the microbiological criteria of final products remains a requirement, such patterns may be very helpful in the development of self-control plans, making the contamination monitoring more realistic [6,8].

**Figure 2:**
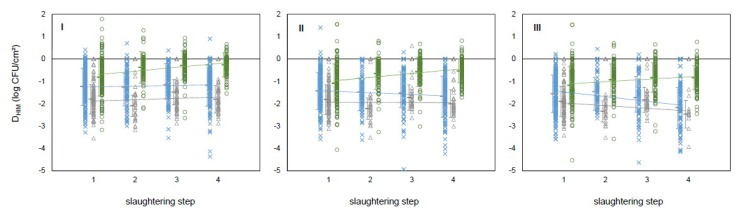
Differences between the microbial counts (log CFU/cm^2^) of MA and a given hygiene indicator microorganism (D_HIM_; I: Enterobacteriaceae; II: Total coliforms; and III: *Escherichia coli*) as a function of the slaughtering step (step 1: Hide after bleeding; step 2: Carcass after hide removal; step 3: Carcass after evisceration; and step 4: Carcass after end washing), and the state of sample collection (blue cross: MG; gray triangle: PR; green circle: RS). The slopes are state-dependent and significantly different from zero (*p* < 0.05) while the error around the linear trend can well be approximated by the normal distribution, with fairly constant deviation (see the error bars around the respective averages).

**Figure 3:**
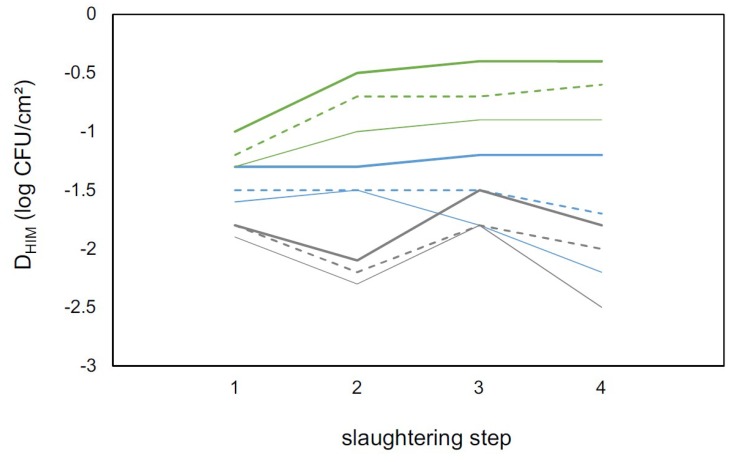
Differences between the microbial counts (log CFU/cm^2^) of MA and a given hygiene indicator microorganism (D_HIM_; Enterobacteriaceae: Dot lines; Total coliforms; dash lines; and *Escherichia coli*: Continuous line) in different steps of slaughtering (step 1: Hide after bleeding; step 2: After hide removal; step 3: After evisceration; and step 4: After end washing) in eight slaughterhouses located in three Brazilian states (Minas Gerais: Blue lines; Paraná: Grey lines; and Rio Grande do Sul: Green lines).

Denoting the differences between the log counts of a given HIM from the correspondent MA by D_EB_, D_TC_, and D_EC_, a linear trend can be established (*p* < 0.05) along the slaughtering steps:

D_HIM_ = *a_HIM_*·step + *b_HIM_* + ε (1)

where the slope *a_HIM_* depends on the state and the ε random component is normally distributed with a deviation 0.6 < σ < 0.8 (Figure 2). The same linear pattern proved to be valid for the differences for D_TC_ and D_EB_ (Figure 3). The averages of the D_HIM_ values follow a monotonic trend with the steps, as a function of the state, and these values at the edges (steps 1 and 4) determine the values at steps 2 and 3 with acceptable accuracy. As a consequence, the intermediate steps could be spared, since they (together with the nature of their randomness) can be described by the linear model based on steps 1 and 4 only (Figure 3).

**Figure 4:**
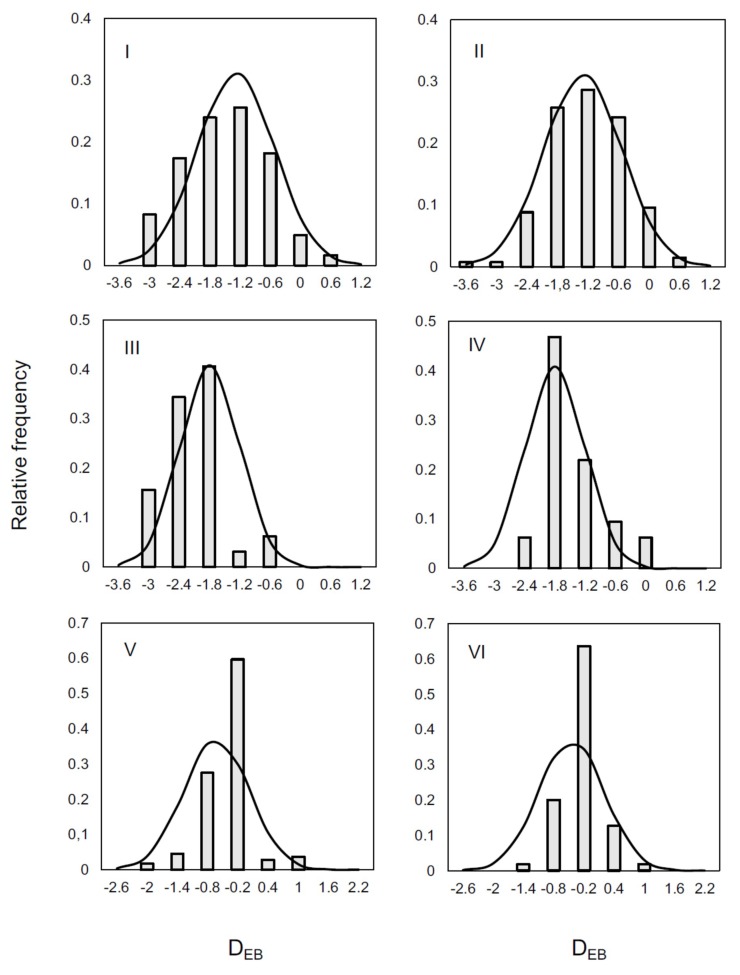
Distribution of D_EB_ values at steps 2 (left) and 3 (right), predicted from D_EB_ data at steps 1 and 4. Minas Gerais: I and II; Paraná: III and IV; and Rio Grande do Sul: V and VI.

For validation, D_EB_ values were modelled by the above linear function (1) using the data from steps 1 and 4 only, then the mean and distribution of D_EB_ at steps 2 and 3 were inferred, assuming normal distribution and deviation, which was the standard error of fitting. The results are summarized in Figure 4, where columns represent the histograms created from the D_EB_ observations. The plots are comparing not only predicted and observed D_EB_ values, but also their standard deviation. The reason why D_EB_ was chosen among the HIM as a reference is that the concentration of EB is generally still high enough that the ‘not-detected’ results do not increase the uncertainty in the data. If the measurements have the same error at low concentrations too, EC would have been a better candidate as the counts of EC and MA encompass the other two (so the interpolation, which is a more robust operation then extrapolation, would be ensured per se). However, analogous trials with the data suggest that D_EB_ is a better choice for modelling than the other two and the D_EB_ results at steps 2 and 3 can be inferred from the respective data at steps 1 and 4 within less than 1 log-unit (0.6–0.8 log CFU/cm^2^) error. The source of error is the inherent variability of the system, shown by the results of replicated measurements. It is likely that the counts of EC and TC can also be inferred from the MA and EB results, as the D_TC_ and D_EC_ look linear depending on the steps, similarly to D_EB_. However, this cannot be unambiguously confirmed due to the bigger errors in the measurements at lower concentrations.

Several factors can affect the microbial counts, as microorganisms are not equally distributed over the carcass. In addition, contamination levels can vary according to several factors such as the day of sampling, sample collection procedure, and analytical method [8,27,40]. Thus, beef carcasses quality assessment requires careful interpretation. Despite the requirement for microbiological analysis in beef carcasses only after final washing (step 4), the systematic analysis of microbiological indicators and pathogens along cattle slaughtering and beef processing is important to provide historical data on the hygiene during processing and check for compliance with holistic quality control programs based on GHP and HACCP [17,34].

Among the sampled beef carcasses, 32 (6.9%) and 71 (15.3%) presented counts above the microbiological criteria established by (EC) No. 1441/2007 for MA and EB, respectively. Most of them were obtained in facilities located in the MG state (Table 4). These results indicate that improvements in slaughter hygiene and a review of process controls are demanded. The monitoring preconized by self-control programs implies costs for the companies. However, identification of specific hygienic weak points along the slaughter process is necessary to improve the quality of final products, and it only can be achieved through microbiological data [6,8,41]. Considering that the EU has set MA and EB as microbiological criteria for beef carcasses [5,18], the counts of these two HIM were selected to design a mathematical model capable of predicting the populations of CT and EC. The proposed model also indicated that the microbiological data obtained at slaughtering steps 1 and 4 were enough to infer the contamination level in intermediary steps 2 and 3, helping the beef industry to develop more objective and feasible quality control programs. Adoption of such programs should have a positive contribution on consumers’ health, enhancing product quality, and enabling companies to maintain and enlarge their markets based on the recognition of their rigorous quality management initiatives.

## Figures and Tables

**Table 1:** Characteristics of the selected Brazilian slaughterhouses.

Slaughterhouse	Location (State) ^1^	Slaughter Rate	Employees	Export
Sl 01	MG	150–180/day	50	Yes
Sl 02	MG	90–100/day^2^	25	No
Sl 03	MG	130–150 /day	50	Yes
Sl 04	PR	600–650/day^3^	40	No
Sl 05	PR	400–450/day	50	No
Sl 06	PR	200–250/day	50	No
Sl 07	RS	130–150/day	140	No
Sl 08	RS	600–650/day	700	Yes

^1^ MG, Minas Gerais; PR, Paraná; and RS, Rio Grande do Sul; ^2^ slaughtering on 2 or 3 days a week; ^3^ slaughtering on 1 or 2 days a week.

**Table 2:** Mean counts (log CFU/cm^2^ ± SD) of hygiene indicator microorganisms (HIM) in samples collected at four steps of the slaughtering process in eight Brazilian slaughterhouses.

HIM	Slaughtering Step			
	1	2	3	4
MA	4.51 ± 0.06 ^a^	2.47 ± 0.06 ^c^	2.64 ± 0.06 ^c^	2.93 ± 0.06 ^b^
EB	3.27 ± 0.06 ^a^	1.79 ± 0.08 ^c^	2.20 ± 0.07 ^b^	2.11 ± 0.07 ^b^
TC	3.20 ± 0.06 ^a^	1.73 ± 0.09 ^c^	2.05 ± 0.08 ^b^	1.81 ± 0.07 ^bc^
EC	3.06 ± 0.06 ^a^	1.58 ± 0.09 ^bc^	1.89 ± 0.08 ^b^	1.53 ± 0.08 ^c^

MA, mesophilic aerobes; EB, Enterobacteriaceae; TC, total coliforms; EC, *Escherichia coli*; slaughtering steps: (1) hide after bleeding; (2) after hide removal; (3) after evisceration; and (4) after end washing. For each microbial group, different letters indicate significant differences among slaughtering steps (*p* < 0.0001).

**Table 3:** Mean counts (log CFU/cm^2^ ± SD) of hygiene indicator microorganisms (HIM) in samples collected at four steps of the slaughtering process, in slaughterhouses located in the states of Minas Gerais (MG), Paraná (PR) and Rio Grande do Sul (RS), Brazil.

HIM	State	Slaughtering Step			
		1	2	3	4
MA	MG	4.66 ± 0.08 ^Aa^	2.85 ± 0.07 ^Ad^	3.30 ± 0.09 ^Ac^	3.8 ± 0.09 ^Ab^
	PR	4.34 ± 0.11 ^Ba^	2.43 ± 0.09 ^Bb^	2.03 ± 0.12 ^Bc^	2.46 ± 0.12 ^Bb^
	RS	4.42 ± 0.09 ^ABa^	2.14 ± 0.07 ^Cb^	2.37 ± 0.09 ^Bb^	2.21 ± 0.1 ^Bb^
EB	MG	3.37 ± 0.08 ^Aa^	1.89 ± 0.1 ^Ac^	2.24 ± 0.11 ^Abc^	2.46 ± 0.08 ^Ab^
	PR	2.56 ± 0.11 ^Ba^	0.84 ± 0.17 ^Bc^	1.45 ± 0.23 ^Bb^	1.02 ± 0.15 ^Cbc^
	RS	3.61 ± 0.09 ^Aa^	2.01 ± 0.1 ^Ac^	2.37 ± 0.12 ^Ab^	2.09 ± 0.1 ^Bbc^
TC	MG	3.25 ± 0.1 ^Aa^	1.77 ± 0.13 ^ABb^	2.05 ± 0.13 ^Ab^	2.0 ± 0.7 ^Ab^
	PR	2.63 ± 0.13 ^Ba^	1.22 ± 0.22 ^Bb^	0.95 ± 0.25 ^Bb^	0.85 ± 0.15 ^Bb^
	RS	3.45 ± 0.1 ^Aa^	1.84 ± 0.12 ^Ac^	2.34 ± 0.13 ^Ab^	1.86 ± 0.08 ^Ac^
EC	MG	3.14 ± 0.1 ^Aa^	1.72 ± 0.12 ^Ab^	1.91 ± 0.15 ^Ab^	1.66 ± 0.1 ^Ab^
	PR	2.46 ± 0.13 ^Ba^	0.93 ± 0.2 ^Bb^	0.86 ± 0.28 ^Bb^	0.52 ± 0.24 ^Bb^
	RS	3.36 ± 0.1 ^Aa^	1.66 ± 0.1 ^Ac^	2.12 ± 0.13 ^Ab^	1.55 ± 0.08 ^Ac^

MA, mesophilic aerobes; EB, Enterobacteriaceae; TC, total coliforms; EC, *Escherichia coli;* slaughtering steps: (1) hide after bleeding; (2) after hide removal; (3) after evisceration; and (4) after end washing. MG, Minas Gerais; PR, Paraná; and RS, Rio Grande do Sul. For each hygiene indicator group, different upper-case letters indicate significant differences among states (*p* < 0.05), and different lower-case letters indicate significant differences among slaughtering steps (*p* < 0.005).

**Table 4:** Number of carcasses after final washing (step 4) with counts above the criteria established by (EC) No. 1441/2007 for mesophilic aerobes (M = 5 log cfu/cm^2^) and Enterobacteriaceae (M = 2.5 log cfu/cm^2^).

State	*n*	Mesophilic Aerobes	Enterobacteriaceae
MG	209	30 (14.4%)	61 (29.2%)
PR	105	2 (1.9%)	1 (1.0%)
RS	150	0 (0.0%)	9 (6.0%)
Total	464	32 (6.9%)	71 (15.3%)

MG, Minas Gerais; PR, Paraná; and RS, Rio Grande do Sul.

## Supplementary Materials

Supplementary materials can be found at https://www.mdpi.com/2076-2607/7/3/86/s1.
