# Supplementary figures and images for: Microbiological Testing for the Proper Assessment of the Hygiene Status of Beef Carcasses
Source: Microorganisms. 2019 Mar 19;7(3):86. doi: 10.3390/microorganisms7030086 (PMC6462900; doi:10.3390/microorganisms7030086)

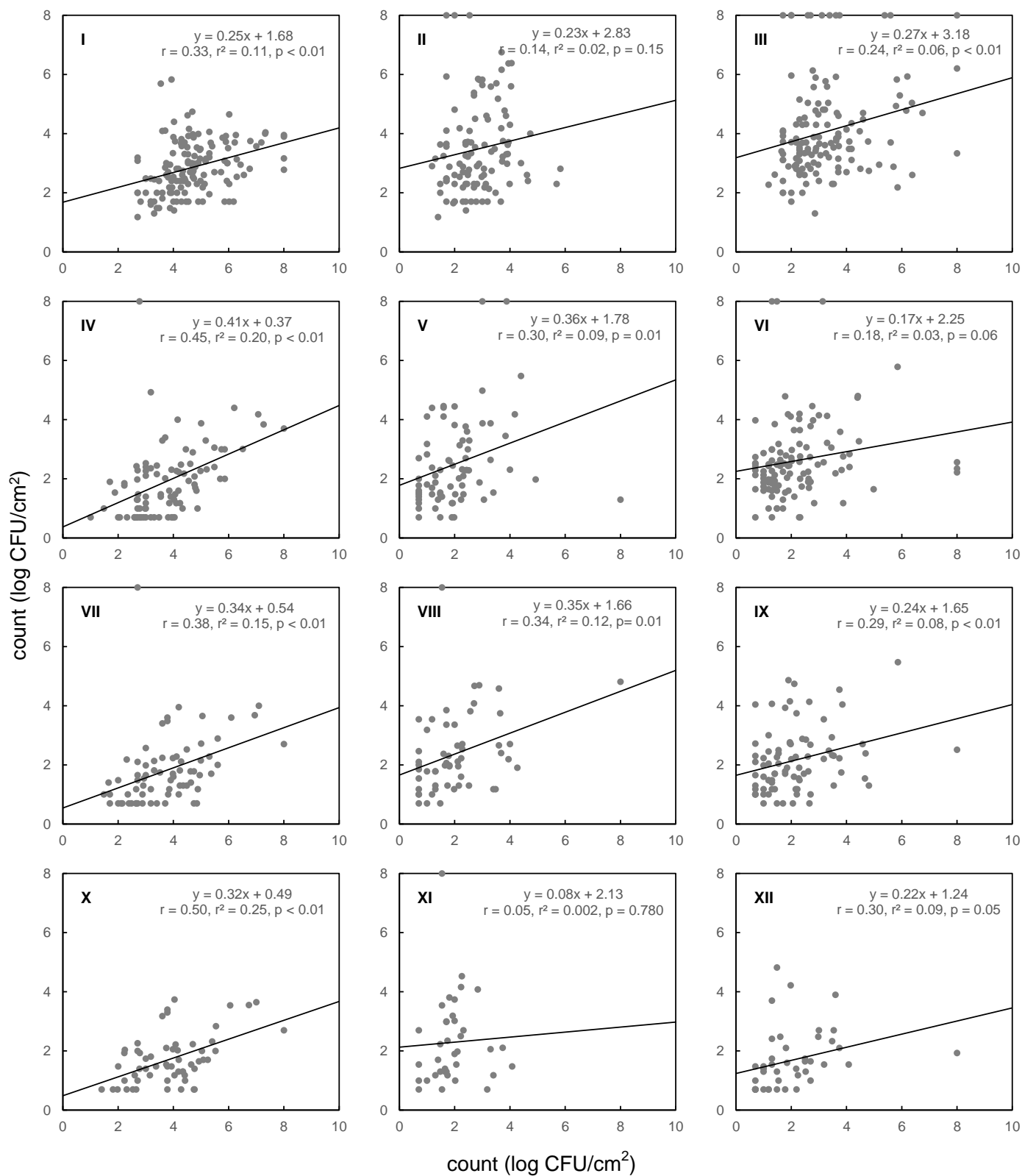

Supplementary Figure 1

Supplement: Supplementary file 1 [file microorganisms-07-00086-s001.zip › Suppl Figure 1 - 2019 01 10.pdf]

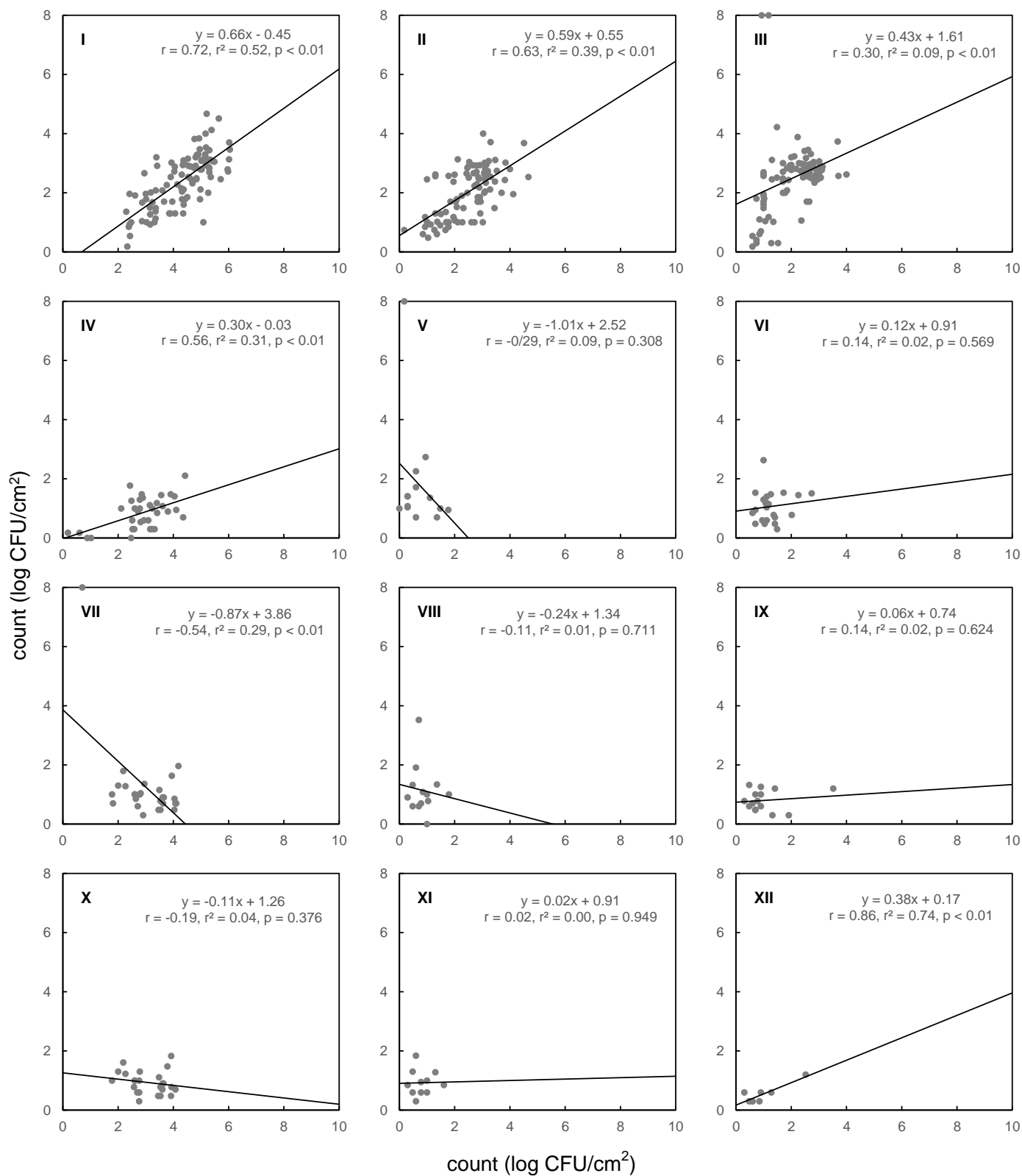

Supplementary Figure 2

Supplement: Supplementary file 1 [file microorganisms-07-00086-s001.zip › Suppl Figure 2 - 2019 01 10.pdf]

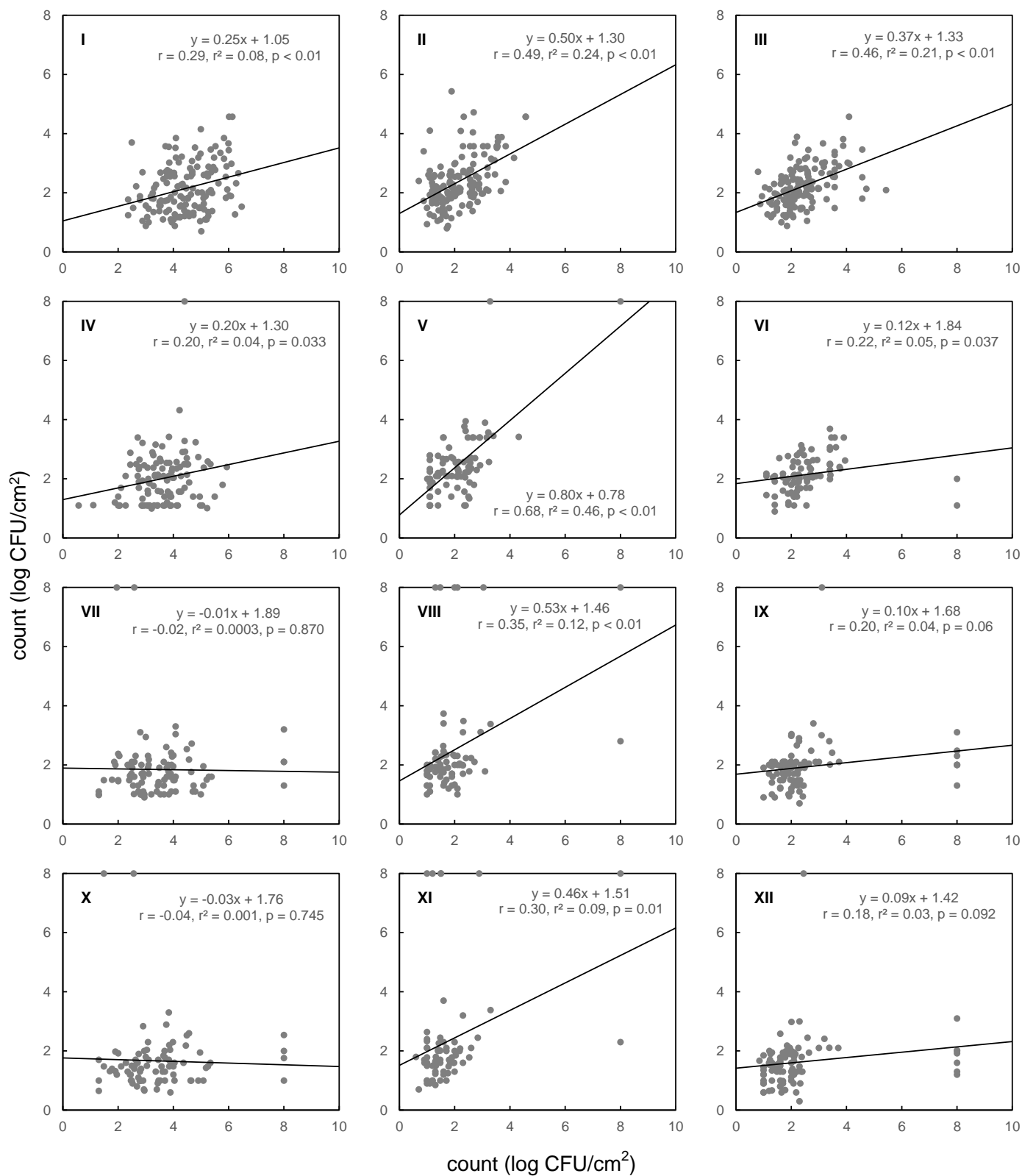

Supplementary Figure 3

Supplement: Supplementary file 1 [file microorganisms-07-00086-s001.zip › Suppl Figure 3 - 2019 01 10.pdf]
